# Supplementary material for: A taxonomic revision of the south-eastern dragon lizards of the Smaug warreni (Boulenger) species complex in southern Africa, with the description of a new species (Squamata: Cordylidae)
Source: PeerJ. 2020 Mar 25;8:e8526. doi: 10.7717/peerj.8526 (PMC7102504; doi:10.7717/peerj.8526)
Supplement: Supplemental Information 3 — Performed using a dataset of 16 morphological characters (three mensural: head length, width and height; 13 meristic: supraciliaries, suboculars, supralabials, infralabials, sublabials, occipitals, gulars, dorsal scale rows transversely and longitudinally, ventral scale rows transversely and longitudinally, femoral pores, subdigital lamellae on fourth toe). [file peerj-08-8526-s003.docx]

**Table S3.** Factor loadings for the Principal Component Analysis of the *Smaug warreni* species complex. Performed using a dataset of 16 morphological characters (three mensural: head length, width and height; 13 meristic: supraciliaries, suboculars, supralabials, infralabials, sublabials, occipitals, gulars, dorsal scale rows transversely and longitudinally, ventral scale rows transversely and longitudinally, femoral pores, subdigital lamellae on fourth toe).

|  | PC1 | PC2 | PC3 | PC4 | PC5 | PC6 |
| --- | --- | --- | --- | --- | --- | --- |
| Head width | 0.52490852 | -0.1710741 | 0.05559668 | 0.12865616 | 0.016943 | -0.1429756 |
| Head length | 0.54078694 | -0.0921106 | 0.05934101 | 0.1049871 | -0.0305539 | -0.110963 |
| Head depth | 0.52933186 | -0.1359806 | 0.06010734 | 0.03614204 | -0.0257305 | -0.0878828 |
| Supraciliaries | 0.0588267 | 0.34568916 | -0.3912365 | -0.1123385 | 0.39173851 | -0.2334951 |
| Subocular | 0.01705864 | -0.0772592 | -0.4253334 | 0.41412495 | 0.09723779 | 0.13882577 |
| Supralabials | 0.02838527 | 0.06757734 | 0.35676653 | 0.03061533 | -0.0576917 | 0.22599264 |
| Infralabials | -0.0144443 | 0.17128476 | 0.07544722 | 0.27719966 | -0.6212453 | -0.110491 |
| Sublabial | -0.1012205 | -0.0975014 | -0.177617 | 0.41243508 | 0.1197748 | 0.20064355 |
| Occipitals | -0.1066507 | -0.0131734 | 0.4511003 | -0.1885027 | 0.20438347 | -0.1467029 |
| Gulars | -0.186098 | 0.07192471 | 0.06327951 | 0.45679843 | 0.06351716 | -0.5046538 |
| Dorsals transversely | 0.05549275 | 0.55820442 | 0.14573891 | -0.0292001 | -0.1537641 | -0.1375286 |
| Dorsals longitudinally | 0.08232249 | 0.49998173 | 0.07016732 | 0.29779211 | 0.08378506 | -0.0746693 |
| Ventrals transversely | 0.05545319 | 0.05328413 | 0.28966016 | 0.06540716 | 0.54528768 | -0.1077314 |
| Ventrals longitudinally | -0.201566 | -0.2573442 | 0.08964652 | 0.00120765 | -0.168776 | -0.4370273 |
| Femoral pores | 0.09522907 | 0.2765288 | 0.19286757 | 0.12506166 | 0.0105519 | 0.51241839 |
| Subdigital lamellae 4th toe | -0.167872 | -0.2498457 | 0.35844294 | 0.43066131 | 0.17081365 | 0.12779712 |
