# Supplementary material for: A taxonomic revision of the south-eastern dragon lizards of the Smaug warreni (Boulenger) species complex in southern Africa, with the description of a new species (Squamata: Cordylidae)
Source: PeerJ. 2020 Mar 25;8:e8526. doi: 10.7717/peerj.8526 (PMC7102504; doi:10.7717/peerj.8526)
Supplement: Supplemental Information 4 — Performed using a dataset of 16 morphological characters (three mensural: head length, width and height; 13 meristic: supraciliaries, suboculars, supralabials, infralabials, sublabials, occipitals, gulars, dorsal scale rows transversely and longitudinally, ventral scale rows transversely and longitudinally, femoral pores, subdigital lamellae on fourth toe). [file peerj-08-8526-s004.docx]

**Table S4:** Standardised coefficients in the linear discriminate analysis of the *Smaug warreni* species complex. Performed using a dataset of 16 morphological characters (three mensural: head length, width and height; 13 meristic: supraciliaries, suboculars, supralabials, infralabials, sublabials, occipitals, gulars, dorsal scale rows transversely and longitudinally, ventral scale rows transversely and longitudinally, femoral pores, subdigital lamellae on fourth toe).

|  | LD1 | LD2 |
| --- | --- | --- |
| Head width | -0.7942333 | -0.2439036 |
| Head length | 0.95772964 | 0.19621842 |
| Head depth | -0.647655 | 0.01202485 |
| Supraciliaries | -0.547079 | 0.67578942 |
| Subocular | -0.404416 | -0.2863855 |
| Supralabials | -0.7575525 | -1.4936172 |
| Infralabials | 0.10451661 | 0.537736 |
| Sublabial | -2.049008 | 1.24066859 |
| Occipitals | -0.0100491 | 1.26944095 |
| Gulars | -0.114057 | 0.1368104 |
| Dorsals transverse | 0.40647081 | -0.1974885 |
| Dorsals longitudinal | 0.17374062 | 0.43889623 |
| Ventrals transverse | -0.3261675 | -0.0130751 |
| Ventrals longitudinal | -0.3143033 | 0.8649316 |
| Femoral pores | 0.15891454 | -0.4237875 |
| Subdigital lamellae 4th toe | -0.1197255 | -0.5015671 |
