# Supplementary figures and images for: A taxonomic revision of the south-eastern dragon lizards of the Smaug warreni (Boulenger) species complex in southern Africa, with the description of a new species (Squamata: Cordylidae)
Source: PeerJ. 2020 Mar 25;8:e8526. doi: 10.7717/peerj.8526 (PMC7102504; doi:10.7717/peerj.8526)

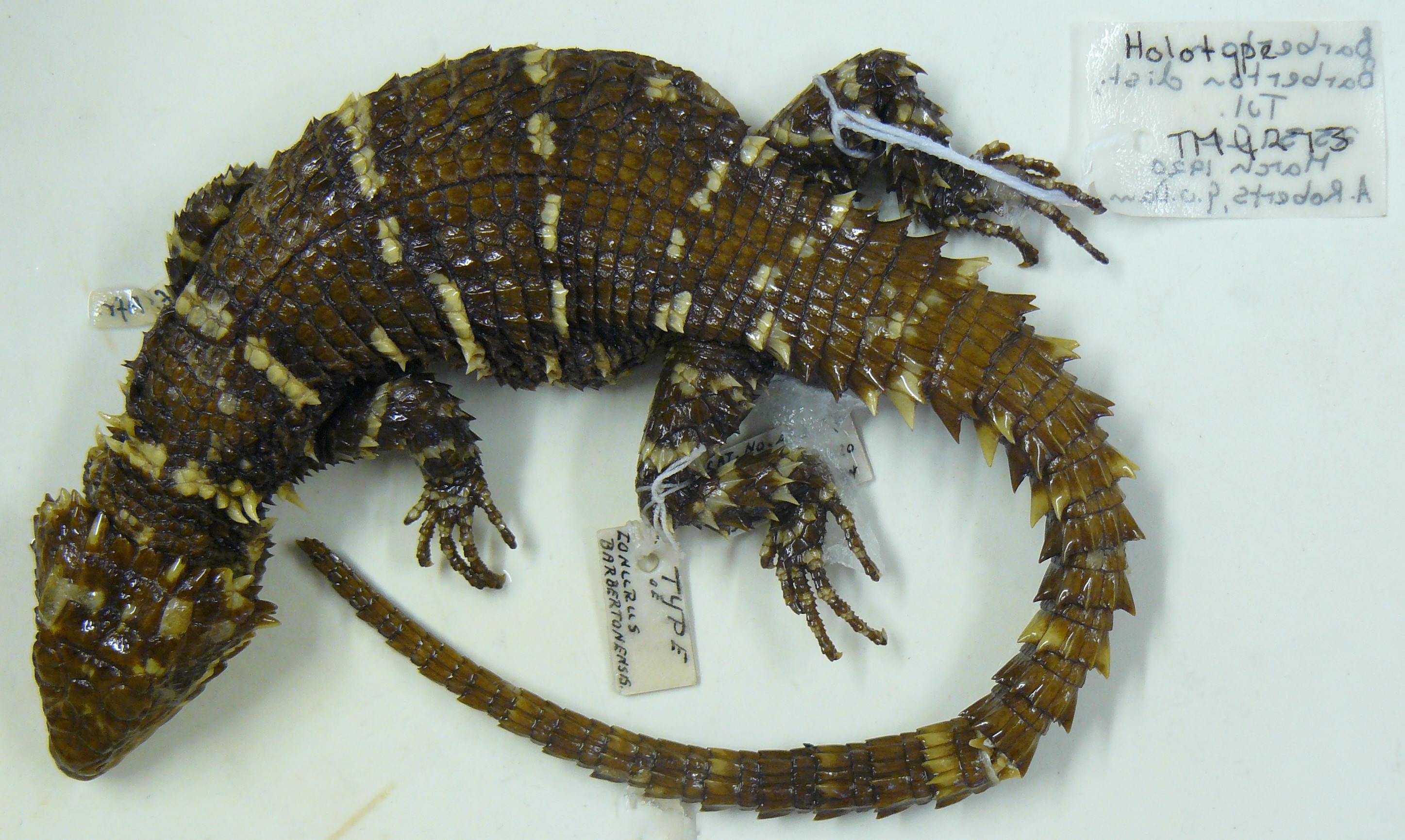

Supplement: Supplemental Information 5 [file peerj-08-8526-s005.jpg]

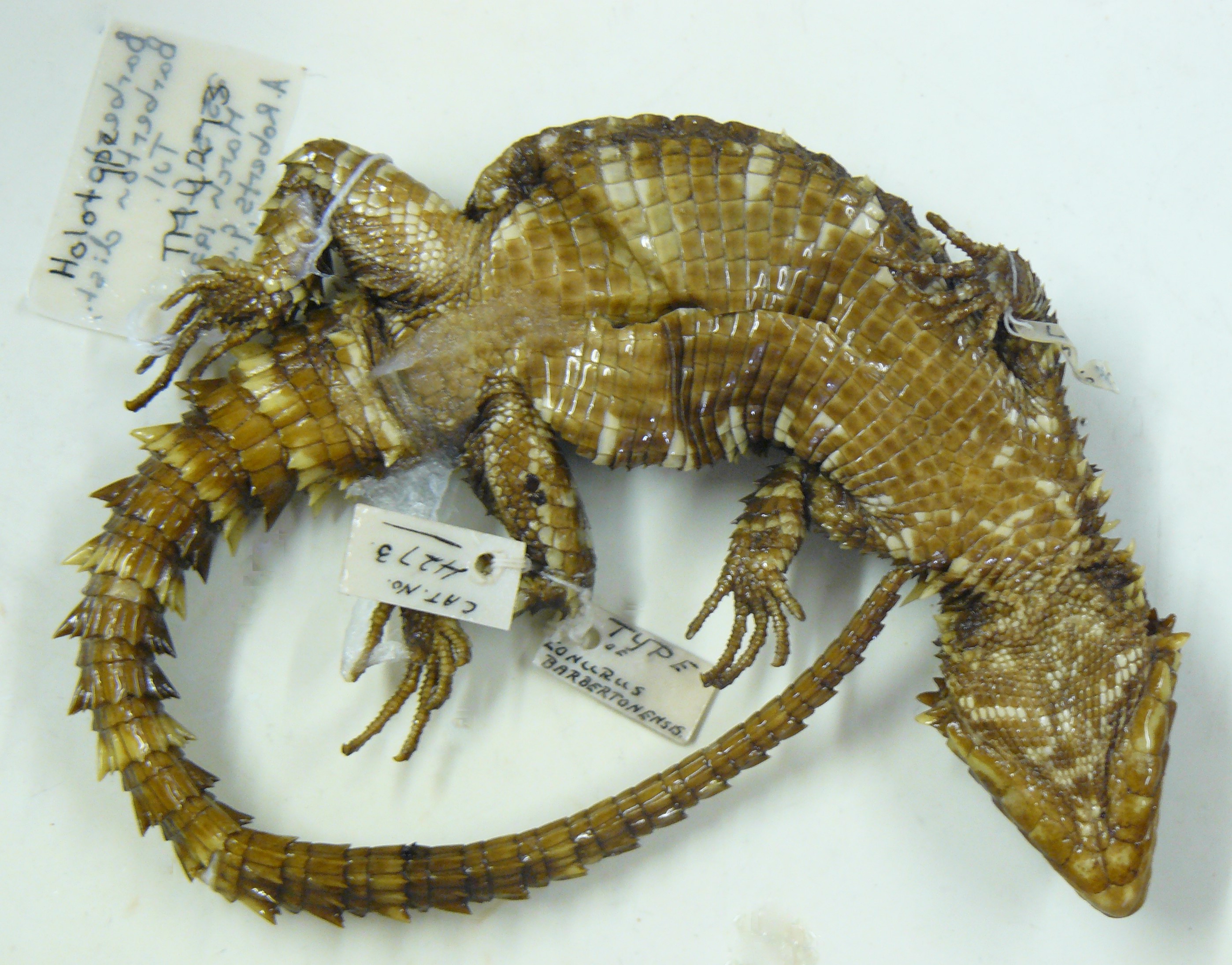

Supplement: Supplemental Information 6 [file peerj-08-8526-s006.jpg]
